# Optimization of CLARITY for Clearing Whole-Brain and Other Intact Organs^1^,^2^,^3^

**DOI:** 10.1523/ENEURO.0022-15.2015

**Published:** 2015-05-25

**Authors:** Jonathan R. Epp, Yosuke Niibori, Hwa-Lin (Liz) Hsiang, Valentina Mercaldo, Karl Deisseroth, Sheena A. Josselyn, Paul W. Frankland

**Affiliations:** 1 Program in Neurosciences and Mental Health, Hospital for Sick Children, Toronto, Ontario M5G 1X8, Canada; 2 Institute of Medical Sciences, University of Toronto, Toronto, Ontario M5G 1X8, Canada; 3 Departments of Psychology and Physiology, University of Toronto, Toronto, Ontario M5G 1X8, Canada; 4 Department of Bioengineering and Psychiatry, Stanford University, Stanford, California 94305

**Keywords:** 3D imaging, CLARITY, light sheet microscopy, neuron morphology, tissue clearing, whole-brain imaging

## Abstract

The development, refinement, and use of techniques that allow high-throughput imaging of whole brains with cellular resolution will help us understand the complex functions of the brain. Such techniques are crucial for the analysis of complete neuronal morphology—anatomical and functional—connectivity, and repeated molecular phenotyping. CLARITY is a recently introduced technique that produces structurally intact, yet optically transparent tissue, which may be labeled and imaged without sectioning. However, the utility of this technique depends on several procedural variables during the process in which the light-scattering lipids in a tissue are replaced by a transparent hydrogel matrix. Here, we systematically varied a number of factors (including temperature, hydrogel composition, and polymerization conditions) to provide an optimized, highly replicable CLARITY procedure for clearing mouse brains. We found that for these preparations optimal tissue clearing requires electrophoresis (and cannot be achieved with passive clearing alone) for 5 d with a combination of 37 and 55°C temperature. Although this protocol is optimized for brains, we also show that it can be used to clear and analyze a variety of organs. Brain or other tissue prepared using this protocol is suitable for high-throughput imaging with confocal or single-plane illumination microscopy.

## Abstract (precis)

CLARITY is a novel technique for optical clearance and intact imaging of biological specimens. This method was introduced in 2013 but has been difficult for some researchers to implement.

## Significance Statement

CLARITY is a novel technique for optical clearance and intact imaging of biological specimens. This method was introduced in 2013 but has been difficult for some researchers to implement. Here, we have optimized the technique to increase the success and consistency of the clearing procedure. Using our protocol, it is possible to clear not only brain, but other tissues as well. In addition, the cleared tissue becomes sufficiently transparent such that no specialized microscopes are necessary to visualize the sample. This optimization of CLARITY will make the technique more widely accessible.

## Introduction

Understanding brain function is a fundamental scientific objective with crucial clinical relevance. Realization of this goal is hampered by the complexity of the brain. There are roughly 86 billion neurons in the human brain (75 million in the mouse) and these neurons interact in complex neural circuits. Using Camillo Golgi’s newly developed techniques to label neurons and their processes, Santiago Ramon y Cajal was among the first to use microscopy to understand brain function. Imaging studies in the tradition of Cajal have been plagued by either poor spatial scale (examining only a small part of a larger brain network) or poor spatial resolution (examining a large network but at the cost of understanding microstructure). The brain has a high lipid content, which makes it opaque and limits the depth of light penetration. Light that does penetrate brain tissue is scattered, producing out-of-focus images in which the point source of the light cannot be localized. Therefore, the high lipid content of the brain makes it especially difficult to image at the mesoscale.

To overcome this obstacle, most experiments involve slicing the brain into relatively thin sections (20-50 µm) before histological staining, imaging, and quantification. However, sectioning the brain has its own drawbacks. For instance, light continues to be scattered even within relatively thin brain sections (limiting axial resolution), sectioning may damage and/or distort the tissue, and much of the rich structural information may be lost in sectioned tissue, even if attempts are made to accurately reconstruct 3D images. For example, sectioned brain tissue may not capture the full extent of neuronal projections (which may extend hundreds of micrometers), thus providing an incomplete view of neuronal branching and regional variations in spine density. One way to address this limitation involves block-face imaging in which a microtome is coupled to a microscope. A tiled image of the brain is acquired prior to cutting each section to produce a 3D reconstruction of the intact tissue (Oh et al., 2014; Vousden et al., 2014). This technique is accompanied by different limitations in that it does not permit immunolabeling of the tissue and requires substantial effort to align the sections after imaging. An ideal method to interrogate tissue is to microscopically examine intact, rather than sectioned, tissue.

Several tissue-clearing protocols have been described to overcome the challenges of imaging thick tissue sections [including SCALE (Hama et al., 2011) and CUBIC (Susaki et al., 2014)] or combinations of organic solvents such as benzyl alcohol/benzyl benzoate (Dodt et al., 2007) or dibenzyl ether (Erturk et al., 2012). Although these methods yield tissue with varying degrees of transparency, they are accompanied by their own limitations. For example, the clearing agents used in these protocols may quench fluorescent proteins and these techniques do not permit antibody labeling. However, a recent technique called lipid-exchanged, anatomically rigid, imaging/immunostaining compatible, tissue hydrogel (CLARITY; Chung et al., 2013) overcomes these limitations to produce transparent tissue without quenching endogenous fluorescence, and this tissue can also be labeled via immunohistochemistry.

CLARITY uses a strong detergent, SDS, to remove lipids from the tissue. To preserve protein integrity and the 3D structure of the tissue, tissue is first perfused with a formaldehyde and acrylamide solution that forms structural hydrogel scaffolding. With the aid of an electrophoretic current, SDS then removes the lipids that are not linked to the scaffold. Cleared tissue may then be labeled and/or imaged with substantially increased light penetration and high-resolution images may be collected several millimeters or more below the tissue surface.

The CLARITY protocol was originally published by the Deisseroth lab (Chung et al., 2013) and methods to optimize the imaging component subsequently added (Tomer et al., 2014). A modified CLARITY protocol, using only passive clearing techniques to avoid the possible tissue damage produced by electrophoretic current, was also recently described (Tomer et al., 2014; Yang et al., 2014; Zhang et al., 2014). However, to effectively take advantage of CLARITY, it is crucial to obtain brains that are highly transparent (for deep imaging) and rigid (to facilitate registration to a reference atlas). While passive clearing may produce transparent tissue, electrophoresis is necessary to achieve the full potential of CLARITY. Here we describe a revised CLARITY protocol designed to fully optimize the clearing process while minimizing concerns related to tissue damage and distortion. Tissue cleared with this protocol can be imaged with virtually any fluorescent microscope, making the technique widely accessible. In addition, we show that our optimized protocol may be used to image numerous intact organs in addition to the brain, with minimal modification.

## Materials and Methods

All of the necessary components and suppliers for performing the procedure can be found in Table 1.

**Table 1:** List of equipment and reagents

Incubating orbital shaker	VWR	97027-346
Vibrating tissue slicer	Leica	VT 1200S
Recirculating water bath	VWR	89203-010
Electrophoresis power supply	Bio-Rad	PowerPac HC
Light sheet microscope	LaVision Biotec	UltraMicroscope
Laser-scanning confocal microscope	Zeiss	LSM 710
Wide-field epifluorescent microscope	Nikon	Eclipse 80i
Vacuum canister	McMaster Carr	2204K7
SDS	Sigma	L3771
Boric acid	Sigma	B6768
Paraformaldehyde	Sigma	441244
Acrylamide solution (40%)	Bio-Rad	161-0140
Bis-acrylamide solution (2%)	Bio-Rad	161-0142
VA-044	WAKO	877-714-1920
Glycerol	Sigma	G5516
Triton-X 100	Sigma	T8787
Sodium azide	Sigma	S2002
10 N NaOH	Sigma	656054
Sodium phosphate monobasic anhydrous	Sigma	S8282
FocusClear	CelExplorer Labs	FC-102
Nitrogen gas	Praxair	
Propidium iodide	Life Technologies	P3566
Clear tubing (PVC, chemical resistant)	McMaster Carr	5103K36
Clear tubing (PVC, chemical resistant)	McMaster Carr	5103K34
Barbed tube fittings (polypropylene)	McMaster Carr	53415K207
Barbed tube fittings (polypropylene)	McMaster Carr	5463K245
Barbed tube fittings (polypropylene)	McMaster Carr	5463K2458
Barbed tube fittings (polypropylene)	McMaster Carr	5463K2457
Barbed tube fittings (polypropylene)	McMaster Carr	5117K51
Manifold (polypropylene)	McMaster Carr	5364K231
Manifold plugs (polypropylene)	McMaster Carr	4515K209
Quick disconnect barbed plug	McMaster Carr	5154K69
Quick disconnect barbed socket	McMaster Carr	51545K63
Filter case	McMaster Carr	4448K35
Filter cartridge	McMaster Carr	4422K61
3M Scotch-weld Epoxy DP270	McMaster Carr	7467A17
Platinum wire	Alfa Aesar	10286
Nalgene 60 ml wide-mouth jar	VWR	36319-547
Cell strainer	Fisher	22363548
Paraffin embedding cassette	VWR	18000-000

### Mice

Adult (6-8 week) WT female mice in a hybrid (F1) genetic background were used for most studies (C57BL/6NTac × 129S6/SvEvTac). In some studies, we also used male and female activity-regulated cytoskeleton-associated protein-targeted recombination (ArcTRAP) in active populations of mice (Guenthner et al., 2013; Jax, B6.129(Cg)-*Arc^tm1.1(cre/ERT2)Luo^*/J) crossed with a fluorescent reporter strain. In the ArcTRAP mice, the tamoxifen-inducible CreER^T2^ protein is driven by endogenous promoter/enhancer elements of the activity-dependent immediate early gene Arc (Lyford et al., 1995). ArcTRAP mice were crossed with a reporter mouse (Jax, B6.Cg-*Gt(ROSA)26Sor^tm14(CAG-tdTomato)Hze^*/ J) in which loxP sequences flank a STOP cassette upstream of a tdTomato reporter. In this way, following systemic tamoxifen injection, cells that are sufficiently active will show Cre-mediated recombination, thereby removing the STOP cassette and allowing expression of tdTomato. A 10 mg/ml solution of 4-hydroxytamoxifen was prepared by first dissolving the drug in 100% ethanol and then suspending the solution in sunflower oil. Mice were injected with a dose of 20 mg/ml. Genotypes were determined by PCR analysis of tail DNA samples as previously described (Guenthner et al., 2013). Mice were bred in-house and group housed (3-5 mice per cage) on a 12 h light/dark cycle with food and water available *ad libitum*. Behavioral experiments were conducted during the light phase of the cycle. All procedures were conducted in accordance with the policies of the institutional Animal Care and Use Committee and conformed to the federal guidelines on the care and use of laboratory animals.

### Hydrogel solution preparation

The hydrogel solution used to perfuse the tissue is composed of four key reagents: acrylamide, bis-acrylamide, formaldehyde, and a thermal initiator (VA-044) in PBS. Different strengths of hydrogels can be produced by varying the concentrations of these reagents. We began with the original hydrogel solution (referred to here as 4:4:0.05) containing final concentrations of 4% acrylamide, 0.05% bis-acrylamide, and 4% formaldehyde. We first prepared a 16% formaldehyde solution by adding 80 g of paraformaldehyde powder to 400 ml of dH_2_O and heated the solution to 60-65°C. The solution was cleared with several drops of 10 N NaOH and the volume was filled to 500 ml. Finally, we filtered the solution and cooled to 4°C. Next, we combined 40 ml of cold 40% acrylamide, 10 ml of cold 2% bis-acrylamide, 40 ml of 16% formaldehyde, 40 ml of 10× PBS, and 220 ml of cold dH_2_O and, finally, 1 g of VA-044 thermal initiator. The solution was kept on ice during preparation and was either used immediately after preparation or divided into 40 ml aliquots and frozen at −20°C for later use. Modified hydrogel solutions were made following the same protocol but with 3% final concentrations of acrylamide and formaldehyde or 0.025% bis-acrylamide.

### Tissue collection for brain samples

Mice were transcardially perfused with ice-cold PBS followed by 25 ml of cold hydrogel solution. The brains were rapidly extracted and submersed in cold hydrogel solution and postfixed in the dark at 4°C for 24 h in 10 ml of the same hydrogel solution.

### Tissue polymerization

To ensure consistent hydrogel polymerization, we removed oxygen from the hydrogel solution by placing the sample vials in a vacuum chamber for 30 min. After vacuuming, the chamber was flooded with pure nitrogen gas to displace oxygen as the chamber was opened and the lids placed on the sample vials. Polymerization of the hydrogel solution depends on the VA-044 thermal initiator, which is triggered by tissue warmed to 37°C. Therefore, we placed the sealed sample tubes in a rotating incubator at 37°C for 3 h. Once the hydrogel was polymerized, excess gel was removed from the brain by gently wiping the gel from the surface with a Kimwipe. We then placed the cleaned brain into a new 50 ml tube containing clearing solution (4% SDS in 0.2 m borate buffer) overnight to wash residual hydrogel solution out of the tissue. The tissue-clearing solution was prepared by dissolving 61.83 g of boric acid and 200 g of SDS in 4.5 L of dH_2_O. The final pH was adjusted to 8.5 using 10 N NAOH.

### Active tissue clearing

**Figure 1:**
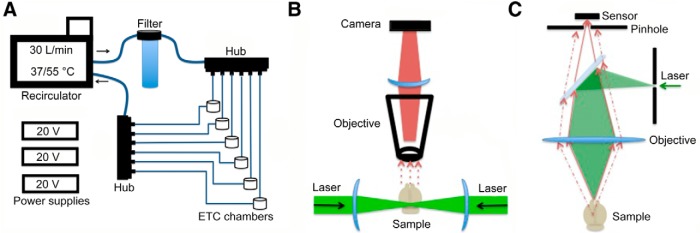
Schematic of electrophoresis and imaging components. Schematic of the electrophoresis system used for active clearing of tissue. ***A***, A recirculating water bath filled with clearing solution is connected via chemical- and heat-resistant tubing to a filter unit (which removes particulate matter and reduces bubbles in the clearing solution). The clearing solution is then split into six parallel tube lines, each containing one ETC chamber. The fluid output of the chambers is then recombined into a single line before entering the recirculator. Power supplies provide current to each ETC chamber. Schematic of the LaVision Biotec UltraMicroscope (***B***) compared with confocal microscope (***C***). The light sheet design permits high-speed image capture of large tissue volumes. In contrast, a confocal microscope may be used for high-resolution imaging of CLARITY tissue, although the acquisition rate is much slower.

We used a modular multichamber design that allowed six brains to be cleared simultaneously using a single recirculator (Fig. 1*A*). Electrophoretic tissue-clearing (ETC) components were connected in parallel to the recirculator with temperature- and chemical-resistant tubing. Clearing solution from the recirculator was first passed through a filter cartridge and then into a hub that divided the solution into six tubing lines connected to the ETC chambers that in turn were connected to another hub that connects back to the recirculator. Power supplies were connected to the platinum leads of the ETC chambers with alligator clips. Small pieces of tissue (such as 2 mm sections or dissected brain regions) were placed into a closed paraffin embedding cassette. Larger tissues were placed into a mesh cell strainer. The cell strainer or cassette was then placed between the electrodes and shielded from direct contact with the electrodes by two sheets of plastic mesh.

Various settings were used for tissue clearing. We have experienced difficulties in regulating the temperature and, as a result, maintaining tissue quality if the clearing voltage is too high or if the recirculator flow rate is too low. Therefore, in all conditions we used a low voltage of 20 V and a high flow rate of 30 L/min (5 L/min/ETC chamber). Under these conditions, the temperature of the clearing solution measured between the electrodes did not fluctuate from the set point. We then varied the temperature between 21 and 55°C and length of clearing from 1 to 5 d to produce the best combination of rigid and clear tissue. The pH of the clearing solution was monitored daily and replaced once the pH dropped from 8.5 to 8.0. After ETC the tissue was rinsed in PBST for several days.

### Passive tissue clearing

For experiments in which tissue was passively cleared we used the same clearing solution and kept the samples on a shaker at 21°C, 37°C, or 55°C. The clearing solution was exchanged every 3 d for up to 2 months. The tissues were then rinsed in several changes of PBST for several days.

### Tissue labeling

Propidium iodide labeling was performed by incubating the tissue for 24-48 h at 37°C in a 1:2000 dilution of propidium iodide in 0.1 m PBST. Immunohistochemistry was performed using 1:100 dilutions of antibody in PBST and incubating the tissues for 7 d at 37°C for the primary antibody and an additional 7 d for the secondary antibody. The tissue was rinsed in 0.1 m PBST after each antibody incubation. The antibodies used were mouse monoclonal anti-smooth muscle actin (Santa Cruz Biotechnology; catalog #SC-53142), rabbit polyclonal anti-collagen type IV (Millipore; catalog #AB756P), rabbit polyclonal anti-CD34 (Santa Cruz Biotechnology; catalog #SC-9095), goat anti-mouse Alexa 488 (Life Technologies; catalog #A11001), and goat anti-rabbit Alexa 488 (Life Technologies; catalog #A11034).

### Refractive index matching

After the PBST washes, the tissue was transferred to either 80% glycerol or FocusClear. The tissue was left to equilibrate in the refractive index matching solution for 24 h at 37°C for glycerol or at room temperature for FocusClear. Finally, the samples were then mounted in the same medium for imaging.

### Imaging setup

In principle, any type of fluorescent microscope can be used to image CLARITY-cleared tissue. However, the microscope must be able to physically accommodate the size of the sample and the stage translation, and the working distance of the microscope objective should be appropriate. A microscope should also allow images to be acquired in a practical length of time. For these reasons, we routinely used light sheet microscopy (LaVision BioTec UltraMicroscope; Fig. 1*B*) for acquisition of large tissues. We also utilized a laser-scanning confocal microscope (Zeiss LSM710; Fig. 1*C*) equipped with 10× NA 0.3 and 25× NA 0.8 objectives and an Epifluorescent microscope (Nikon Eclipse 80i) with a 10× NA 0.3 objective

### Image analysis

We used a Lenovo ThinkStation D30 with 2× Intel Xeon 3.1 GHz processors, a QuadK5000 4 GB graphics card, and 128 GB of RAM. For the light sheet microscope, images were acquired using the ImSpector software suite (LaVision Biotec), confocal images were acquired using Zen (Zeiss), and wide-field images were acquired with NIS Elements (Nikon). Image processing was then performed using a combination of software packages including Imaris (Bitplane) for 3D reconstruction, ImageJ for 2D stitching and basic adjustments, and AutoQuant (Media Cybernetics) for deconvolution.

### Statistical analysis

**Figure 2:**
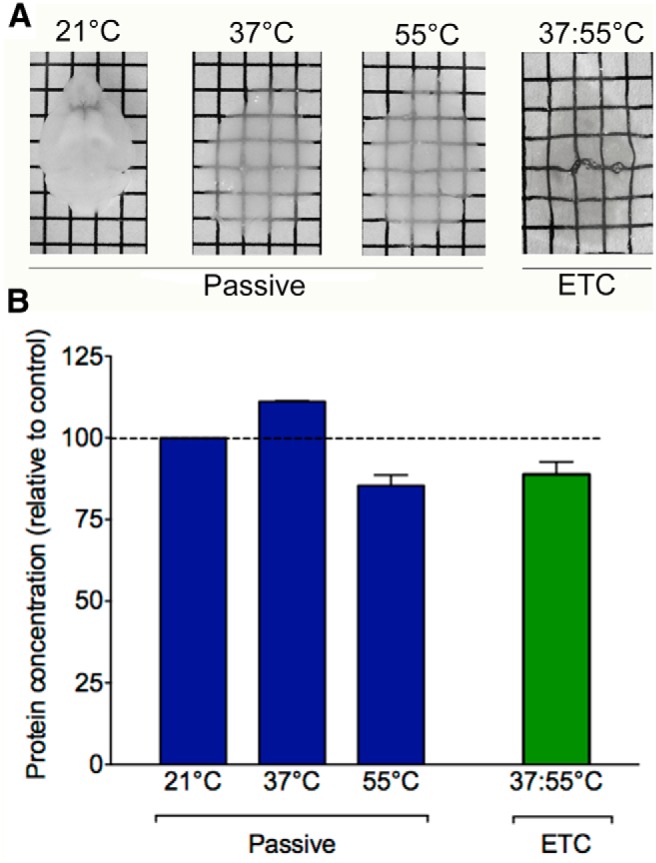
Active clearing of brains with ETC is more efficient than passive clearing. ***A***, Passive clearing is temperature dependent with very little clearing at 21°C occurring after 1 month in SDS. At 37 and 55°C the brain becomes progressively clearer after 1 month in SDS but does not get as clear as with active (ETC) clearing. ***B***, Relative to freshly fixed and uncleared tissue, protein content is well preserved following both active and passive clearing methods, regardless of temperature used. Data are shown relative to brains that were perfused and polymerized with the same hydrogel solution but were not cleared. Olfactory bulb tissue from each clearing condition was homogenized and a BCA assay was performed to estimate protein concentration. There were no significant differences in protein concentration as an effect of active versus passive clearing (*p* = 0.4) or clearing temperature (*p* = 0.24).

**Figure 3:**
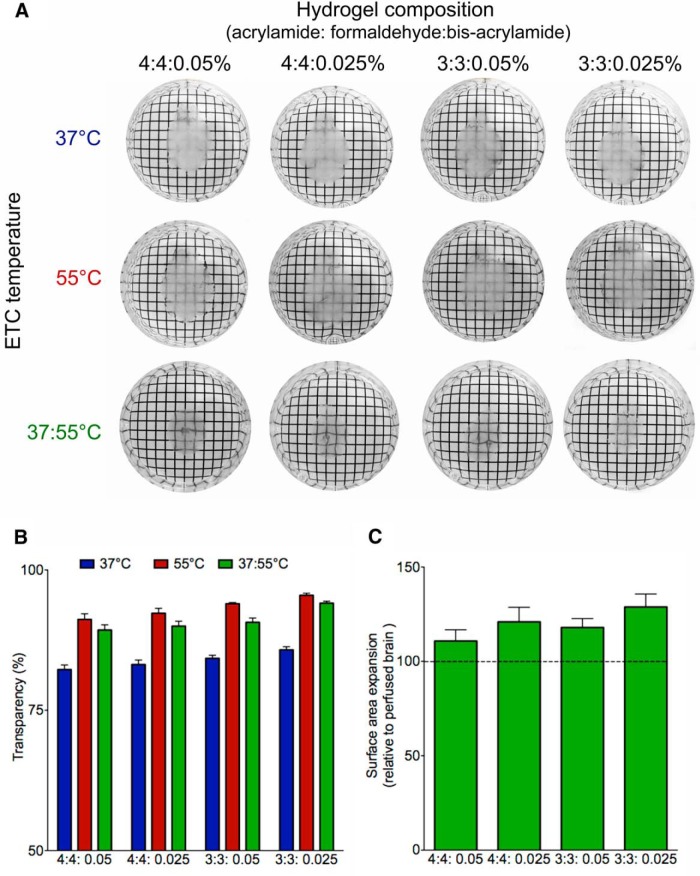
Clearing conditions for optimal transparency. ***A***, Comparison of ETC temperature and hydrogel composition on tissue transparency and expansion. There were significant main effects of clearing temperature (*F*_(2,24)_ = 178.9, *p <* 0.00001) and hydrogel concentration (*F*_(3,24)_ = 16.2, *p <* 0.00001) on transparency Tissue appears clearest with 37/55°C ETC and 3% acrylamide, 3% formaldehyde, and 0.025% hydrogel. Tissue expansion is less in the 37/55°C condition compared with either 37°C or 55°C alone. ***B***, Both 55 and 37/55°C produce tissue that is more transparent than 37°C, regardless of hydrogel composition (*p*s = 0.0002 and 0.0009, respectively). Transmission was measured on a light table under standardized illumination conditions. A reference measurement was made without a sample and was set to 100%. The sample was then imaged on the light table and the percentage light intensity through the specimen was recorded as a percentage of the reference value. ***C***, Brains cleared with the combined 37/55°C clearing protocol expand more in lower concentration hydrogel (3% formaldehyde:3% acrylamide:0.025% bis-acrylamide compared with higher concentration hydrogel (4% formaldehyde:4% acrylamide:0.05% bis-acrylamide). The conditions for producing an optimal combination of stable and clear tissue is to polymerize the tissue with hydrogel composed of 4% acrylamide, 4% formaldehyde, and 0.05% bis-acrylamide and then clear the tissue with 5 d of ETC (4 d at 37°C and 1 d at 55°C).

Data shown in Figures 2 and 3 represent the mean ± SEM. Analyses were conducted using two-way ANOVAs and Newman–Keuls *post hoc* tests as described in Table 2.

**Table 2:** Data analysis

Data	Test	*n*	Main effect	*F*	*P*
BCA analysis	Main effects ANOVA	Three per group	Active versus passive temperature	0.791.68	0.400.24
Transparency	Two-way ANOVA	Three per group	HydrogelTemperatureInteraction	16.20178.900.70	0.0000060.0000000.63
	Newman–Keuls*Post hoc*	Three per group	Hydrogel 3:3:0.025 versus 3:3:0.05 3:3:0.025 versus 4:4:0.025 3:3:0.025 versus 4:4:0.05 3:3:0.05 versus 4:4:0.05Temperature37°C versus 37/55°C37°C versus 55°C37:55°C versus 55°C		0.0047 0.00023 0.00017 0.00530.00015 0.000093 0.00013

## Results

### Active versus passive tissue clearing

Tissue may be cleared passively or facilitated with electrophoresis (ETC) using custom electrophoretic chambers. Electrophoresis decreases the amount of time required to produce clear tissue. Passive clearing is less expensive as no additional equipment is required. In terms of effectiveness of the clearing process, our comparisons demonstrate that although passive clearing can be somewhat effective, it is not as effective as ETC.

Temperature remains a critical factor in passively clearing tissue. The efficacy of the clearing procedure increases with temperature. At 21°C, passive clearing has little effect even after 1 or 2 months in SDS solution. However, at 37°C, moderately clear tissue can be achieved in 1 month. At a higher temperature of 55°C, very clear tissue can be produced in 1 month. However, after 1 month, passively cleared tissue is not nearly as clear as tissue cleared with ETC (Fig. 2). Faster passive clearing times may be achieved by using a lower concentration of acrylamide, formaldehyde, or bis-acrylamide, but at the potential cost of decreased tissue preservation and increased tissue distortion.

### Optimal hydrogel composition

The basic hydrogel solution is composed of formaldehyde (to act as a fixative), acrylamide (to form the hydrogel), bis-acrylamide (to act as a cross-linker), and VA-044 (as a thermal initiator). The original publication listed the solution as 4% formaldehyde, 4% acrylamide, and 0.05% bis-acrylamide. The structural rigidity and porosity of the hydrogel are a function of the concentration of these reagents. Figure 3 shows the effect of varying concentrations of acrylamide and bis-acrylamide on tissue expansion and clearing. If the goal is to examine an endogenous fluorescent marker, we recommend using the 4% formaldehyde and 4% acrylamide mixture to ensure the tissue structure is well preserved. Conversely, if the goal is to perform immunohistochemistry on the tissue, we recommend using a hydrogel composition with 3% acrylamide and 3% formaldehyde to facilitate antibody penetration.

### Optimal clearing time and temperature

**Figure 4:**
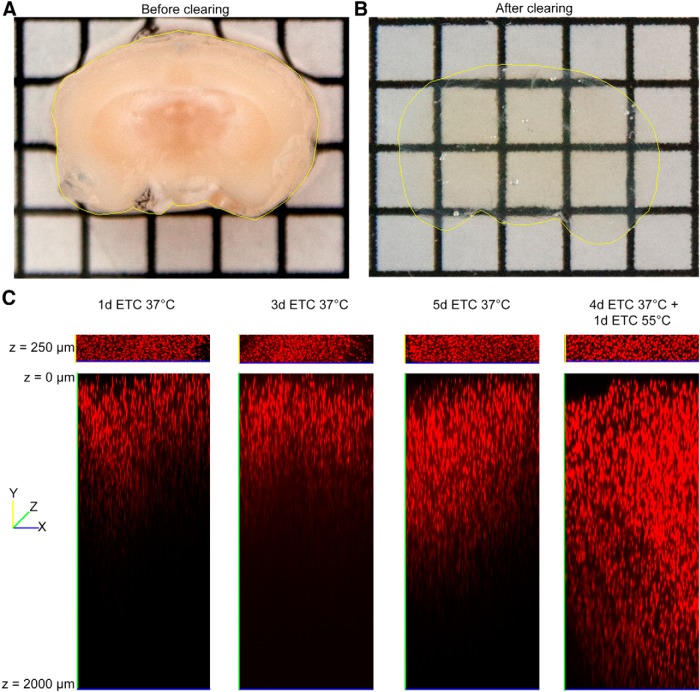
The effect of ETC duration and temperature on imaging depth. A 2mm thick coronal section before (A) and after (B) clearing with out optimized protocol. C, Coronal mouse brain section (2 mm thick) cleared with ETC for 1 d, 3 d, or 5 d at 37°C or for 4 d at 37°C plus an additional day at 55°C. Sections were subsequently stained with propidium iodide and imaged using a confocal microscope under identical excitation conditions without the use of *Z*-attenuation correction. There is an increase in depth at which a fluorescent signal may be imaged with respect to clearing protocol. The 37/55°C clearing temperature provides deepest imaging penetration.

**Figure 5:**
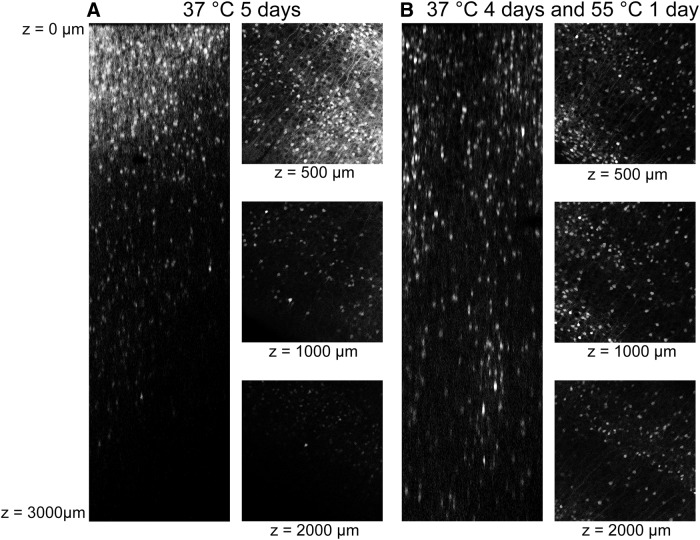
Imaging depth optimization. Using ArcTRAP-tdTomato mice we visualized the endogenous fluorescence signal in mice brains that underwent 5 d of ETC at 37°C (***A***) or were cleared for 4 d at 37°C followed by 1 d at 55°C (***B***). Improved imaging depth is observed using the combined 37/55°C protocol, suggesting that the improvement relates to light rather than dye penetration.

Running the ETC at 20 V for 5 d produces optimal results for clearing intact mouse brains. For the first 4 d, the temperature of the clearing solution was kept at 37°C. On the final day, the temperature is increased to 55°C to complete the clearing. In our experience, this pulse of higher temperature produces more transparent brain tissue than is produced by running the ETC at 37°C alone. Conversely, while running ETC at 55°C for several days produces very clear tissue, the brain tends to lose structural integrity (Fig. 3). The transparency of the tissue increases during ETC over the course of 5 d. After 1-3 d of clearing, the imaging depth of the brain is limited to several hundred micrometers, but we observed a noticeable increase in the imaging depth following 5 d of ETC at 37°C. A far greater increase in imaging depth of at least 2 mm is achieved in a period of 5 d if the final day of clearing is conducted at a higher temperature of 55°C (Fig. 4). The improved imaging ability following our combined 37/55°C clearing protocol could result from either increased penetration of the propidium iodide or increased tissue transparency and deeper light penetration (although both are desirable effects). While we cannot eliminate the possibility that the propidium iodide was better able to penetrate the tissue following more complete clearing, we can demonstrate that our optimal clearing protocol does greatly enhance the depth at which an endogenous fluorescent marker can be imaged (Fig. 5). Therefore, the improved imaging depth is primarily a result of improved transparency of the tissue.

### Glycerol versus FocusClear for refractive index matching

**Figure 6:**
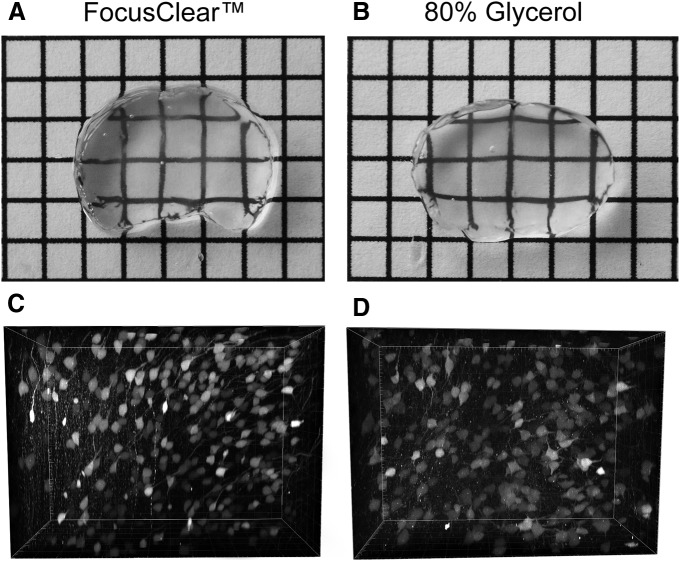
Examining effects of different imaging media (FocusClear vs glycerol). Refractive matching of clarified tissue with either FocusClear or glycerol produces equally transparent tissue, and high-quality images can be collected using either medium. ***C***, ***D***, We imaged the tdTomato signal in the same sections shown in ***A*** and ***B***. Excellent image clarity is produced whether the tissue is placed in FocusClear or 80% glycerol.

After clearing, the tissue must be transferred to an imaging medium with a refractive index that matches that of the tissue. Several options are available. FocusClear and BrainClear are commercially available solutions, which are preferred by several groups. Another option is to use 80% glycerol as the immersion solution. Here we compared the efficacy of glycerol with FocusClear by incubating cleared 3-mm-thick coronal brain sections in either medium. We observed little difference between FocusClear and 80% glycerol other than cost (Fig. 6). Excellent quality images can be acquired in both mediums and similar imaging depth is achievable. Therefore we routinely use 80% glycerol as our preferred refractive index matching solution.

### Optimized CLARITY can be imaged without specialized microscopes

**Figure 7:**
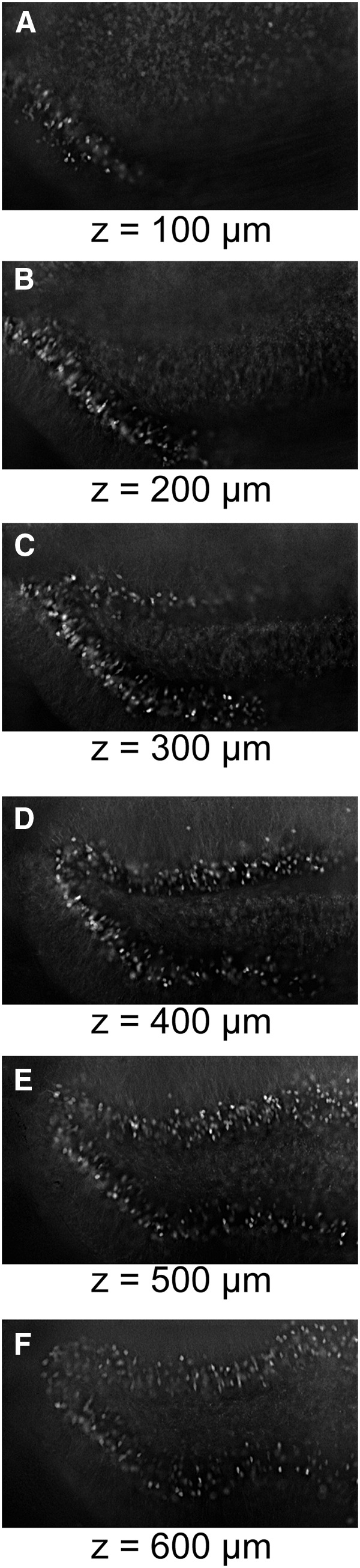
Examining effects of different microscopes to image clear brains. Although a light sheet or confocal microscope may be ideal, when sufficiently clear, tissue may be imaged using even a very basic epifluorescent microscope. In this case, a Nikon Eclipse 80i with a mercury arc lamp was used to image a 2-mm-thick cleared section. ***A–F***, Single image planes at 100 µm intervals through the *z*-stack. Bright and sharp cells can be seen through at least 600 µm of the tissue.

Our optimized CLARITY protocol produces extremely clear tissue with preserved morphology and fluorescence that can be used for various applications, including anatomical pathway tracking or brain-wide activity mapping. In principle, subsequent imaging may be conducted on a variety of different microscopes. Based on the excitation mechanisms used by each type of microscope, we believe that light sheet microscopy is optimal for fast imaging of large tissue volume with confocal or multiphoton imaging as slower alternatives. However, not all laboratories have access to these types of microscopes. Therefore, we tested whether wide-field epifluorescent microscopy could be used to image CLARITY-cleared tissue. In this study, we exposed an activity-reporter mouse (ArcTRAP; Guenthner et al., 2013) to a novel context. We then perfused the mouse with a hydrogel solution, sectioned the brain into 2-mm-thick sections, and cleared the tissue. Figure 7 shows an image stack collected manually (5 µm *z*-steps) on a Nikon eclipse epifluorescent microscope equipped with a mercury arc lamp and 10× 0.3 NA air objective. An iterative, blind deconvolution algorithm was run on the resulting image stack. The tdTomato signal can be clearly visualized through at least 600 µm of the 2 mm slice. Although a more powerful microscope allows higher resolution imaging, these images show that an epifluorescent microscope may prove adequate for tasks such as cell quantification in thick tissue sections. The simple addition of a motorized stage would also allow for low-cost automated acquisition of thick tissue sections on a very basic microscope.

### Application of CLARITY for imaging neuronal morphology

**Figure 8:**
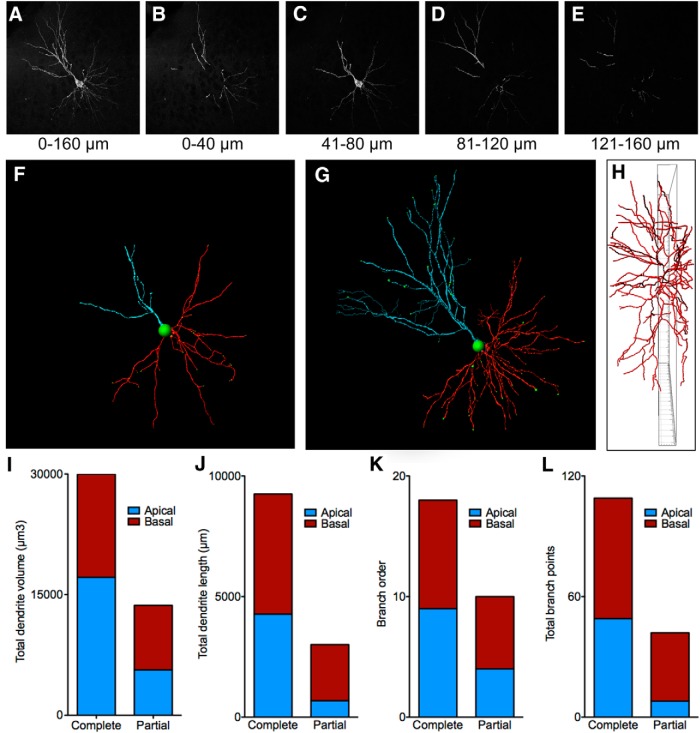
Using CLARITY to analyze neuron morphology. ***A***, GFP-labeled cortical pyramidal neuron visualized in a CLARITY-processed tissue section (HSV-GFP viral vector). ***B–E***, The same neuron shown with the *Z*-depth limited to consecutive 40-µm-thick hypothetical sections. Quantification of dendritic processes based on any individual 40 µm section will produce an incomplete representation of a complex neuron. ***F***, A 40-µm-thick slice of the same neuron centered on the soma is shown. ***G***, A tracing of the entire neuron is shown. ***H***, The neuron is rotated around the *y*-axis to show how much of the dendritic arbor would be lost using standard section thickness. The inner frame is 40 µm thick. ***I–L***, Example data automatically collected using Imaris from the apical and basal dendrites of the traced neuron. The complete neuron was compared with a single 40-µm-thick portion of the neuron centered on the cell body.

**Figure 9:**
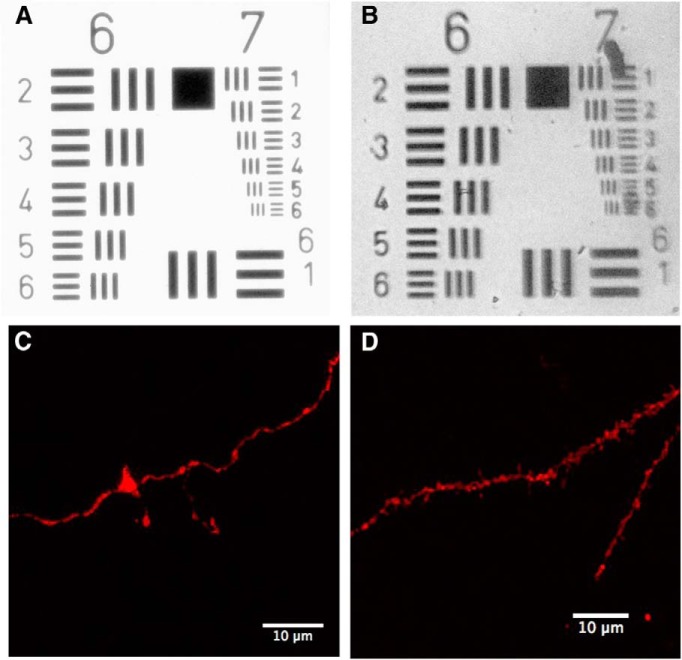
Image resolution in cleared tissue. ***A***, The USAF test target pattern imaged with a 10× air objective on a Nikon light microscope. The lines in element 6-6 and 7-6 are ∼4.4 and 2.2 µm thick, respectively. ***B***, The same test pattern is shown with a cleared brain (∼6 mm thick) over the pattern. The 2.2-µm-thick lines can still be resolved through the tissue. ***C***, Analysis of dendritic spines is possible in cleared tissue using a confocal microscope with 25× oil objective. ***D***, Axonal filopodia in the mossy fiber pathway can be observed in thick tissue sections.

The complexity and size of many types of neurons makes it difficult to fully analyze their morphology using standard histology approaches. Golgi staining can be used to analyze branching and spine densities in relatively thick tissue sections, but this technique is difficult to reliably combine with immunohistochemistry for a more detailed analysis. Imaging of fluorescently labeled neurons allows for combined morphological analysis with immunohistochemistry, but without tissue clearing the thickness of the section that can be used is quite limited. As a result, the analysis of the neuronal morphology is typically based on only a portion of the entire neuron or complex and time-consuming reconstructions are required. Imaging neurons in cleared tissue is ideal as the entire neuron can be visualized without physical reconstruction. In addition to the information that is gained in the *z*-axis, CLARITY produces images with a high signal-to-noise ratio due to the transparent nature of the tissue. This makes CLARITY ideal for automating the analysis of neuronal morphology in intact tissue. We demonstrate these capabilities by imaging a complete GFP-labeled pyramidal neuron and automatically detecting and reconstructing the neuron with the Imaris Filament tracer module. Information regarding the 3D nature of the neuron can quickly be assessed including branching patterns, dendrite lengths, and volumes. Figure 8, A–L (see also Movie 1), illustrates the extent of the neuron that may be missed by standard histology (using a 40-µm-thick tissue section). As can be seen in this image, both the size of the dendritic arbor and the branch order are greatly underestimated if analysis is confined to a 40-µm-thick substack. In addition, differences in the ratio between apical and basal dendrites may be observed when observing the entire neuron rather than a portion of it. An important consideration may be the resolution and resulting accuracy with which neurons can be reconstructed using CLARITY versus serial sections. We have not performed an exhaustive comparison of neurons imaged using CLARITY compared with those reconstructed from serial sections. However, the resolution obtained while imaging in deep tissue appears sufficient for accurately quantifying spines and axonal filopodia. Ultimately, the resolution that is achievable is a factor of the imaging setup; however, even with a low-cost 10× air objective on an epifluorescent microscope, a resolution of at least 2 µm can be achieved at a depth of ∼6 mm (Fig. 9).

Movie 1 A complete neuron infected with HSV-GFP. The complete neuron, which is traced in red, is compared with a 50 µm subsection of the neuron centered on the cell body shown in blue. 10.1523/ENEURO.0022-15.2015.video.1

### Application of CLARITY for imaging other tissue types

**Figure 10:**
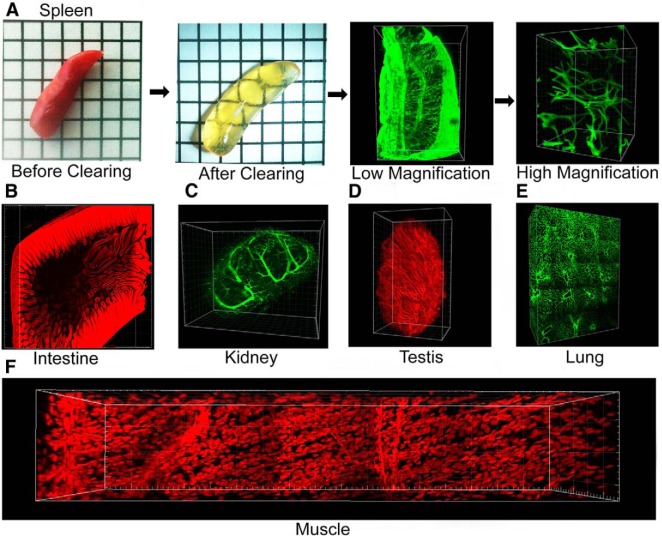
Using CLARITY to clear and image other organs. ***A***, Mouse spleen before and after CLARITY and low- and high-magnification images of CD34 immunohistochemistry on cleared tissue. ***B***, Cleared mouse intestine stained with propidium iodide. ***C***, Cleared mouse kidney labeled for smooth muscle actin. ***D***, Mouse testis stained with propidium iodide. ***E***, Mouse lung immunolabeled for collagen. ***F***, Section of mouse muscle stained with propidium iodide. All fluorescent images were collected using a LaVision Biotec light sheet microscope except for the high-magnification image of the spleen, which was captured using a Zeiss confocal microscope.

CLARITY was originally described for the analysis of brain tissue. However, other organs can also be cleared and imaged using the same protocol. The present clearing protocol can be used to successfully clear spinal cord, intestine (Movie 2), lung (Movie 3), spleen, intestine, testis (Movie 4), kidney, adrenal gland, skin, and muscle. Little modification is required to clear most other organs (Fig. 10). However, some tissues, such as liver, are more difficult to clear with this method. If the tissue is still quite opaque after the first 4 d of ETC at 37°C, it may be necessary to extend the time at this temperature for an additional 1 d or 2 d before switching to 55°C. Further troubleshooting information is provided in Table 3.

**Table 3:** CLARITY troubleshooting

Problem	Solution
The tissue is not clearing	Closely monitor the pH of the clearing solution to ensure it remains between 8 and 8.5. The pH of the solution will decrease over time and solution may needto be replaced during the clearing process. Small volume recirculators will need to be changed more frequently than those that hold a large volume of SDS.
The tissue is “melting”	High temperatures for a prolonged period of time may lead to melting. Ensure that the clearing solution temperature is appropriate. It may be necessary to increase the flow rate of the recirculator to maintain a suitable temperature in the chambers.
The tissue is turning dark yellow or orange	This may be due to high clearing temperature (see above). A localized burning/color change may be the result of the tissue directly contacting an electrode. Tissue should be in close apposition to, but not directly touching, the electrodes. A plastic mesh divider should be placed between the tissue and electrode to prevent direct contact.
The brain has a black residue on the surface	Ensure the cleanliness of the recirculator and filter. A large buildup of black residue on the platinum electrodes can be cleaned by reversing the polarity of the electrodes and running the ETC chambers for several hours. Always ensure that as much hydrogel as possible is removed from the surface of the tissue before beginning the clearing procedure as the hydrogel has a greater tendency to gather residue than the tissue itself.
Cleared tissue is turning opaque in glycerol	On occasion, tissue may become opaque following transfer into 80% glycerol. This can be corrected by returning the tissue back to PBST at 50°C until clear, typicallya few hours. The tissue can then be transferred back to glycerol. This issue seems to be the result of incomplete tissue clearing.
An opaque precipitate is forming in the tissue in FocusClear	A seemingly irreversible black precipitate may begin to form in the tissue if it is left for prolonged periods of time in FocusClear. Tissue should not be stored in FocusClear any longer than necessary to equilibrate and image the tissue.

Movie 2 An image stack showing the 3D structure of a mouse intestine labeled with propidium iodide. 10.1523/ENEURO.0022-15.2015.video.2

Movie 3 A fly-through reconstruction of the vasculature of the mouse lung after staining for collagen. 10.1523/ENEURO.0022-15.2015.video.3

Movie 4 A 3D reconstruction showing a mouse testis labeled with propidium iodide. 10.1523/ENEURO.0022-15.2015.video.4

## Conclusions

CLARITY, when optimized, provides a powerful method for producing transparent and stable tissues that can be stained and imaged in their entirety. We have shown two functional metrics that demonstrate the degree of clearing that is accomplished with our protocol. First, we are able to image the cleared brains using a simple epifluorescent microscope. Second, once the tissue is adequately clear it is possible to use glycerol as a refractive index matching solution rather than FocusClear.

**Figure 11:**
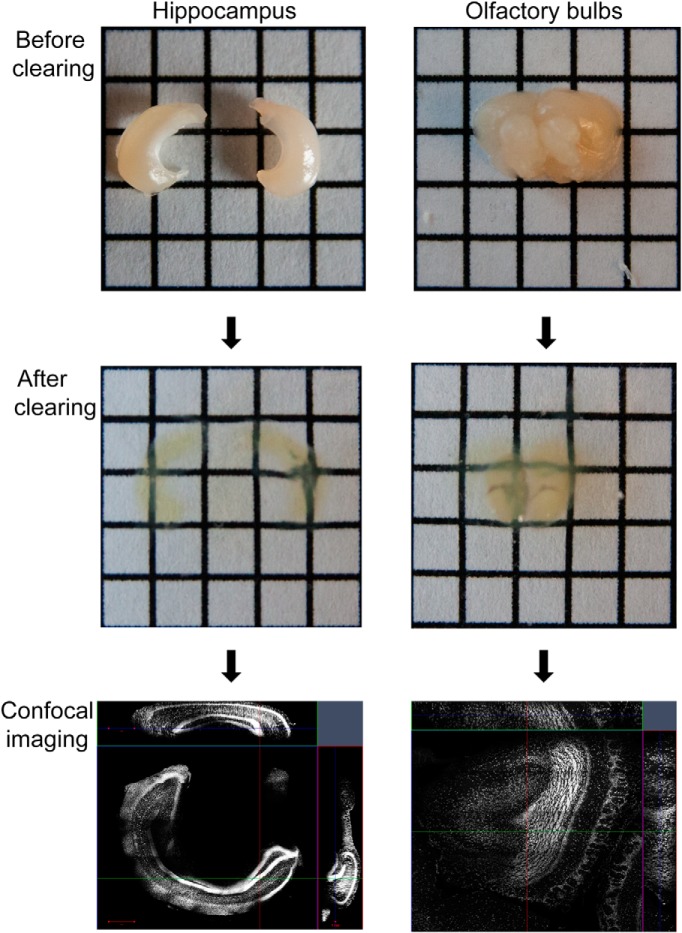
Dissection and clearing of brain subregions. As it may not be necessary to clear the entire brain, it is more efficient to dissect the region of interest before clearing and imaging. Examples of dissected hippocampus and olfactory bulbs before and after clearing and subsequently stained with propidium iodide are shown. Electrophoretic tissue clearing was performed for 3 d (2 d at 37°C and 1 d at 55°C). Imaging of the cleared tissue was performed with a Zeiss 710 confocal microscope and 10× objective.

Our methods for producing consistently clear tissue are based on the following principles. (1) If it is not necessary or possible to image a complete piece of tissue or brain then it is faster and simpler to section or dissect a subregion of the tissue before the clearing process (Fig. 11). (2) It is best to use a structurally rigid hydrogel solution when perfusing the tissue. This ensures that the tissue will be maximally preserved and undergo minimal transformation during the clearing, staining, and imaging steps of the procedure. (3) To completely clear the tissues in a reasonable amount of time, active clearing with ETC is necessary. (4) Potential structural damage to the tissue can be prevented by carefully maintaining the temperature during ETC, using low voltage and preventing any contact between the tissue and electrodes. (5) To create extremely clear tissue, we find the best ETC procedure is to run the samples at 37°C for 4 d followed by a fifth day with the temperature increased to 55°C. One day of clearing at 55°C does not appear to have any negative effects on the tissue and has the positive effects of finishing the clearing more effectively and also helping to reduce tissue expansion.

## Synthesis

The decision was a result of the Reviewing Editor Liisa Galea and the peer reviewers coming together and discussing their recommendations until a consensus was reached. A fact-based synthesis statement explaining their decision and outlining what is needed to prepare a revision is listed below.

Two reviewers and the reviewing editor have read this method paper on optimization of the CLARITY technique. While there are a number of methodological papers in the literature on CLARITY this paper provides some very important straightforward tips with analyzed data to support their optimized protocol. All reviewers thought this paper would provide the research community with valuable information.

The minor issues that need to be addressed are:

1) Figure 4 is used to illustrate the improved clarification with longer incubation time and a 55 deg pulse. In this experiment it is not immediately clear if optical clarification is the sole determinant of the dramatic effect that is shown, or whether there is an effect on propidium iodide penetration. The authors should present a convincing argument that penetration is not an issue and/or address the possibility that propidium iodide penetration may be at play here (or if they have available already use tissue with an endogenous fluorescent protein to make this point).

2) Figure 7 contains some hypothetical sections to make the point that information is lost by examining 40 micron sections rather than thicker tissue slabs. This point is fairly self-evident. However, a more valuable comparison could be made between neurons reconstructed from several 40 um sections and those traced in a single thick slab. For example is the full dendritic tree accurately quantifiable in a thick cleared slab of brain tissue? is it possible that reconstructing from cut sections might reveal detail that a thick section cannot, or that while GFP+ etc cells can be visualized 100s of microns deep they may not be able to be quantified with perfect accuracy? This would involve more work than the authors are interested in doing at this point, and is not critical to the conclusions of the manuscript or the acceptance of this manuscript. However, the authors may wish to discuss this important point or address the caveat that this has not be empirically tested (e.g. actual quantification and comparison with other methods would be required to address adequately address this but is not required in the revision). It does appear that in Figure 7 the authors have quantified one neuron using thick vs hypothesized thin sections in Figures I-L - but it is not clear in figure caption or in text on page 15 if the 'partial' calculation is the summation of all the 40 micron sections? Clarification would be welcome.

3) Since the purpose of this manuscript is to facilitate use of the CLARITY technique it would be useful to include vendor and catalog information for some of the items (eg just as was done for the antibodies, which is appreciated). For example, vacuum chambers, the recirculator, the filter, hubs - what was used and what works?

## Author Response

1) Figure 4 is used to illustrate the improved clarification with longer incubation time and a 55 deg pulse. In this experiment it is not immediately clear if optical clarification is the sole determinant of the dramatic effect that is shown, or whether there is an effect on propidium iodide penetration. The authors should present a convincing argument that penetration is not an issue and/or address the possibility that propidium iodide penetration may be at play here (or if they have available already use tissue with an endogenous fluorescent protein to make this point).

We have addressed this point in the text of the manuscript (page 13). In addition, we have added a new figure (figure 5) which illustrates the effect of our optimized clearing temperature using tissue with an endogenous fluorescent protein. Compared to brains cleared for 5 days at 37°C our improved procedure shows greater imaging depth due to improved penetration of light. This can be seen in 2 ways. One, there is a decrease in background light scattering in the superficial regions of the tissue and second, greater signal from deeper areas of the tissue.

2) Figure 7 contains some hypothetical sections to make the point that information is lost by examining 40 micron sections rather than thicker tissue slabs. This point is fairly self-evident. However, a more valuable comparison could be made between neurons reconstructed from several 40 um sections and those traced in a single thick slab. For example is the full dendritic tree accurately quantifiable in a thick cleared slab of brain tissue? is it possible that reconstructing from cut sections might reveal detail that a thick section cannot, or that while GFP+ etc cells can be visualized 100s of microns deep they may not be able to be quantified with perfect accuracy? This would involve more work than the authors are interested in doing at this point, and is not critical to the conclusions of the manuscript or the acceptance of this manuscript. However, the authors may wish to discuss this important point or address the caveat that this has not be empirically tested (e.g. actual quantification and comparison with other methods would be required to address adequately address this but is not required in the revision).

This is an important concern which, in practice, is a difficult assessment to make because the same neurons cannot be visualized with both normal histology methods and with CLARITY. We have addressed this caveat in the manuscript on page 15 as suggested by reviewers. Although we cannot fully address the question of accuracy we have added an additional figure (figure 9) to help clarify the resolution and level of detail that we obtain using our clearing protocol.

It does appear that in Figure 7 the authors have quantified one neuron using thick vs hypothesized thin sections in Figures I-L - but it is not clear in figure caption or in text on page 15 if the 'partial' calculation is the summation of all the 40 micron sections? Clarification would be welcome.

The partial calculation is based on a single 40µm thick slice centered on the cell body rather than on a reconstruction because this is similar to approaches commonly performed in the literature. In this case the, reconstruction would ultimately yield the same data as for the full neuron because we are dissecting out a subset of optical sections from the complete image stack. We have clarified this in the figure caption.

3) Since the purpose of this manuscript is to facilitate use of the CLARITY technique it would be useful to include vendor and catalog information for some of the items (eg just as was done for the antibodies, which is appreciated). For example, vacuum chambers, the recirculator, the filter, hubs - what was used and what works?

We have added a table (table 1) listing the required equipment and reagents for performing our modified CLARITY protocol.
